# Tunicamycin Mediated Inhibition of Wall Teichoic Acid Affects *Staphylococcus aureus* and *Listeria monocytogenes* Cell Morphology, Biofilm Formation and Virulence

**DOI:** 10.3389/fmicb.2018.01352

**Published:** 2018-07-02

**Authors:** Xingyue Zhu, Dongqi Liu, Atul K. Singh, Rishi Drolia, Xingjian Bai, Shivendra Tenguria, Arun K. Bhunia

**Affiliations:** ^1^Molecular Food Microbiology Laboratory, Department of Food Science, Purdue University, West Lafayette, IN, United States; ^2^College of Science, Nanjing University of Aeronautics and Astronautics, Nanjing, China; ^3^Department of Comparative Pathobiology, Purdue University, West Lafayette, IN, United States

**Keywords:** tunicamycin, wall teichoic acid, *Staphylococcus aureus*, *Listeria monocytogenes*, biofilm, antibiotic resistance, bacterial virulence, optical scatter patterns

## Abstract

The emergence of bacterial resistance to therapeutic antibiotics limits options for treatment of common microbial diseases. Subinhibitory antibiotics dosing, often aid in the emergence of resistance, but its impact on pathogen’s physiology and pathogenesis is not well understood. Here we investigated the effect of tunicamycin, a cell wall teichoic acid (WTA) biosynthesis inhibiting antibiotic at the subinhibitory dosage on *Staphylococcus aureus* and *Listeria monocytogenes* physiology, antibiotic cross-resistance, biofilm-formation, and virulence. Minimum inhibitory concentration (MIC) of tunicamycin to *S. aureus* and *L. monocytogenes* was 20–40 μg/ml and 2.5–5 μg/ml, respectively, and the subinhibitory concentration was 2.5–5 μg/ml and 0.31–0.62 μg/ml, respectively. Tunicamycin pre-exposure reduced cellular WTA levels by 18–20% and affected bacterial cell wall ultrastructure, cell membrane permeability, morphology, laser-induced colony scatter signature, and bacterial ability to form biofilms. It also induced a moderate level of cross-resistance to tetracycline, ampicillin, erythromycin, and meropenem for *S. aureus*, and ampicillin, erythromycin, vancomycin, and meropenem for *L. monocytogenes*. Pre-treatment of bacterial cells with subinhibitory concentrations of tunicamycin also significantly reduced bacterial adhesion to and invasion into an enterocyte-like Caco-2 cell line, which is supported by reduced expression of key virulence factors, Internalin B (InlB) and *Listeria* adhesion protein (LAP) in *L. monocytogenes*, and a *S. aureus* surface protein A (SasA) in *S. aureus*. Tunicamycin-treated bacteria or the bacterial WTA preparation suppressed NF-κB and inflammatory cytokine production (TNFα, and IL-6) from murine macrophage cell line (RAW 264.7) indicating the reduced WTA level possibly attenuates an inflammatory response. These results suggest that at the subinhibitory dosage, tunicamycin-mediated inhibition of WTA biosynthesis interferes with cell wall structure, pathogens infectivity and inflammatory response, and ability to form biofilms but promotes the development of antibiotic cross-resistance.

## Introduction

Extensive and indiscriminate use of antibiotics in the community, hospitals, and clinics have fueled the crisis of antibiotic resistance (Coe et al., 1992). About 80% of all antibiotics sold in the United States are administered to food animals, primarily as a growth promoter and (or) for controlling infection (Marshall and Levy, 2011). As a result, bacteria are often exposed to a subinhibitory (non-lethal) dose of antibiotics. This has played a critical role in the emergence of antibiotic resistance (Andersson and Hughes, 2014), selection for antibiotic-resistant bacteria (Gullberg et al., 2011), and the emergence of multidrug-resistant (MDR) bacterial pathogens, such as extended-spectrum β-lactamase (ESBL) Gram-negative bacteria (Nikaido, 2009; Carlet et al., 2012; Capita and Alonso-Calleja, 2013; Blair et al., 2014). Such emergence of antibiotic resistance in the bacterial pathogens has become a global concern posing a major threat to the public health and livestock (Davies and Davies, 2010; Beceiro et al., 2013; Rodriguez-Rojas et al., 2013). A very few literature exist that correlates the effect of non-lethal dose of antibiotics on the bacterial physiology, virulence, and antibiotic resistance profile. Therefore, in this study, we used nucleoside antibiotic tunicamycin, an antibiotic (870 Da), produced by *Streptomyces* species and is inhibitory towards Gram-positive bacteria (Takatsuki et al., 1971). Tunicamycin inhibits wall teichoic acid (WTA), an important cell wall molecule in Gram-positive bacteria that plays a major role in physiology and pathogenesis.

We used *Staphylococcus aureus* and *Listeria monocytogenes* as model Gram-positive bacterial pathogens to study the effect of WTA-targeting tunicamycin on cell structure, morphology, antibiotic cross-resistance, biofilm formation, and pathogenic attributes. *S. aureus* is a Gram-positive coccus and causes skin and soft tissue infections in both humans and animals (King et al., 2006), leading to serious illnesses, like life-threatening sepsis, endocarditis, pneumonia, meningitis, urinary tract infection, osteomyelitis, arthritis and enteritis (Han et al., 1999; Fowler et al., 2005; Bocchini et al., 2006; Powers and Wardenburg, 2014). It is also one of the common foodborne pathogens and is responsible for over 240,000 foodborne illnesses annually (Scallan et al., 2011). A subpopulation of *S. aureus* is MRSA, which is a major public health concern since it can be hospital-acquired, community-acquired or animal acquired (Kadariya et al., 2014). *L. monocytogenes* is a Gram-positive invasive opportunistic foodborne pathogen and kills more than 5,000 people per year globally with underlying conditions. The mortality rate is about 20% and can be as high as 50%. Infants, the elderly, pregnant women and the patients receiving immunosuppressive drugs or suffering from immunosuppressive viral diseases are most susceptible to this infection (Vazquez-Boland et al., 2001; Radoshevich and Cossart, 2018). Therefore, the effect of WTA-inhibiting tunicamycin at the subinhibitory concentration was studied on these pathogens, which are of clinical and public health importance.

Peptidoglycan and WTA play important role in bacterial physiology and pathogenesis in Gram-positive bacterial pathogens (Schröder et al., 2003; Swoboda et al., 2010; Bucher et al., 2015; Babina et al., 2017). Therefore, to gain a deeper understanding of WTA-targeting antibiotic tunicamycin on bacterial physiology and pathogenesis in situations where optimal antibiotics levels are not maintained, we investigated the effect of subinhibitory concentration of tunicamycin on two model pathogens, *S. aureus* and *L. monocytogenes*. We examined cell morphology, cell wall ultrastructure, bacterial cross-resistance to other antibiotics, biofilm formation, adhesion and invasion to enterocytes, and inflammatory response. We also analyzed the expression levels of cell wall-associated key adhesion protein, LAP (*Listeria* adhesion protein) (Burkholder and Bhunia, 2010; Jagadeesan et al., 2010; Drolia et al., 2018) and invasion proteins, InlA (Internalin A) (Gaillard et al., 1991), and InlB (Internalin B) (Braun et al., 1997; Bierne and Cossart, 2007) in *L. monocytogenes*, and SasA (*Staphylococcus aureus* surface protein A: 240 kDa), a major MSCRAMM (microbial surface components recognizing adhesive matrix molecules) in *S. aureus* (Clarke and Foster, 2006). We observed that pre-exposure of these two pathogens to tunicamycin at subinhibitory concentrations lowered bacterial ability to form a biofilm, expression of key virulence proteins and subsequent bacterial adhesion, invasion, and inflammatory response, but showed the development of moderate cross-resistance to select antibiotics.

## Results

### Minimal Inhibitory Concentration (MIC) and Subinhibitory Dose of Tunicamycin

**FIGURE 1:**
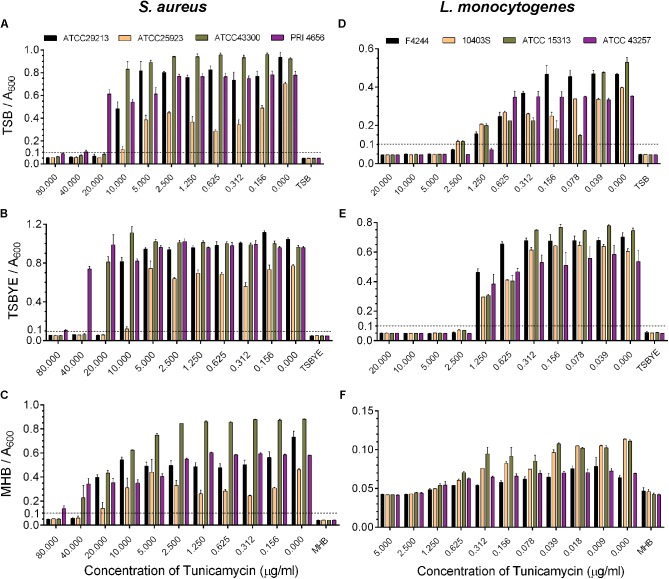
Analysis of minimum inhibitory concentration (MIC) of tunicamycin against four strains of *S. aureus* **(A–C)** and *L. monocytogenes* **(D–F)** in different broth media, tryptic soy broth (TSB) **(A,D)**, TSB with 0.6% yeast extract (TSBYE) **(B,E)**, Mueller Hinton broth (MHB) **(C,F)** in microtiter plates. The absorbance of each blank broth medium was 0.045 ± 0.005 and based on this value, an absorbance > 0.1 was considered positive for growth of cultures in the microtiter plate wells. Three independent experiments were performed to calculate the MIC and errors were represented as the standard error of the mean (SEM).

To establish the role of WTA in antibiotic resistance, and pathogenesis, it is important first to establish the MIC values of tunicamycin, which will be the basis for determining the non-lethal (subinhibitory) dose of tunicamycin. MIC of tunicamycin was tested against four strains of *S. aureus* and *L. monocytogenes* in three bacterial growth media, tryptic soy broth (TSB), TSB containing 0.6% yeast extract (TSBYE), and Muller-Hinton broth (MHB) to find an optimal medium to perform further experiments; however, the MIC values varied (**Figure 1**). The MIC for *S. aureus* strains in TSB, TSBYE and MHB varied from 20 – 40 μg/ml, 20 – 80 μg/ml, and ≥40 μg/ml, respectively, while for *L. monocytogenes* 2.5 – 5 μg/ml, 2.5 μg/ml, and 2.5 μg/ml, respectively (**Figure 1**). Furthermore, *L. monocytogenes* growth was substantially lower in MHB than TSB, hence TSB was chosen for all future experiments.

**FIGURE 2:**
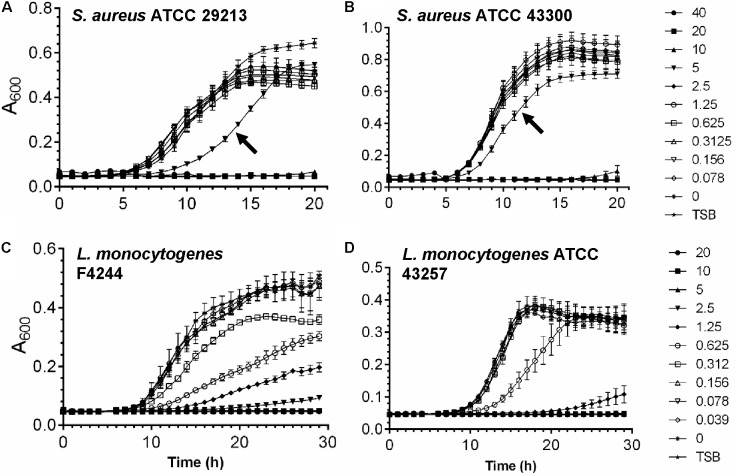
Determination of subinhibitory concentration of tunicamycin for *S. aureus* **(A,B)** and *L. monocytogenes* **(C,D)** in tryptic soy broth (TSB). Graphs showing growth of *S. aureus* ATCC 29213 **(A)** and *S. aureus* ATCC 43300 **(B)** in the presence of tunicamycin at 0–40 μg/ml and growth of *L. monocytogenes* F4244 **(C)** and *L. monocytogenes* ATCC 43257 **(D)** in the presence of tunicamycin at 0–20 μg/ml.

To determine the subinhibitory dose of tunicamycin, two strains of *S. aureus* and *L. monocytogenes* were treated with different concentrations of antibiotic below the MIC dosage for each pathogen, and bacterial growth (Absorbance 600 nm) was monitored over a 20 – 30 h period (**Figure 2**). At 5 μg/ml tunicamycin, *S. aureus* ATCC 29213 and ATCC 43300 growth were significantly slower than when tunicamycin was used below 5 μg/ml or no antibiotic control. However, at higher concentration (≥10 μg/ml), no growth was observed during the 20 h incubation time window for both strains (**Figures 2A,B**). In case of *L. monocytogenes* F4244 and ATCC43257, at 0.625 μg/ml or higher, the bacterial growth rate was significantly lower than when grown at <0.625 μg/ml or no antibiotic control (**Figures 2C,D**). Taken these data together, the subinhibitory concentration of tunicamycin for *S. aureus* was determined to be 2.5–5 μg/ml, and for *L. monocytogenes* 0.312–0.625 μg/ml, and these concentrations were used to study bacterial cell morphology, cross-resistance to other antibiotics, physiology, biofilm-forming abilities and virulence attributes.

### Tunicamycin Pretreatment Enhances Moderate Levels of Bacterial Cross-Resistance to Other Antibiotics

**FIGURE 3:**
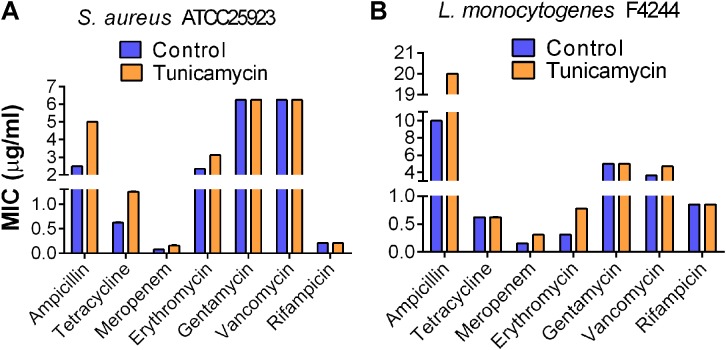
Determination of onset of cross-resistance in tunicamycin pre-exposed *S. aureus* ATCC 25923 **(A)** and *L. monocytogenes* F4244 **(B)** against ampicillin, tetracycline, meropenem, erythromycin, gentamycin, vancomycin, and rifampicin. Control, no tunicamycin; Tunicamycin, bacteria were grown in the presence of a sub-inhibitory concentration of tunicamycin (2.5 μg/ml for *S. aureus*; 0.31 μg/ml for *L. monocytogenes*) for 24 h. MIC values were obtained from two experiments performed in triplicate.

We examined the development of antibiotic cross-resistance in pathogens after 24-h exposure to the subinhibitory concentrations of tunicamycin by estimating the MIC against gentamycin, erythromycin, vancomycin, rifampicin, ampicillin, tetracycline, and meropenem using broth dilution method (Andrews, 2001). The MIC for the control *S. aureus* ATCC 25923 strain (unexposed to tunicamycin) was 2.5 μg/ml for ampicillin, 0.63 μg/ml for tetracycline, 0.08 μg/ml for meropenem, 2.34 μg/ml for erythromycin, 6.25 μg/ml for gentamycin, 6.25 μg/ml vancomycin, and 0.21 μg/ml for rifampicin. While the same strain pre-exposed to 2.5 μg/ml tunicamycin, exhibited about a twofold higher MIC to ampicillin (MIC = 5 μg/ml), tetracycline (MIC = 1.25 μg/ml) and meropenem (MIC = 0.16 μg/ml) and slightly higher MIC to erythromycin (MIC = 3.13 μg/ml) (**Figure 3A**). There were no changes in MIC to gentamycin, vancomycin, and rifampicin. Likewise, tunicamycin (0.31 μg/ml) exposed *L. monocytogenes* F4244 showed twofold increased cross-resistance to ampicillin (MIC = 20 μg/ml), meropenem (MIC = 0.31 μg/ml) and erythromycin (MIC = 0.78 μg/ml) compared to the control (unexposed to tunicamycin) with MIC of 10 μg/ml for ampicillin, 0.16 μg/ml for meropenem and 0.31 μg/ml for erythromycin. The MIC to vancomycin was slightly increased from 3.65 to 4.69 μg/ml after tunicamycin pre-exposure, and MIC to tetracycline (0.63 μg/ml) and rifampicin (0.85 μg/ml) remained unchanged with or without tunicamycin pre-exposure (**Figure 3B**). Bacteria can be induced to develop antibiotic resistance by repeated subculture in the presence of a particular antibiotic under *in vitro* condition. Cross-resistance can develop in such resistant variants against the antibiotics of the similar chemical structure. Cross-resistance among unrelated antibiotics with dissimilar chemical structure and mode of action is not fully understood (Sutherland et al., 1964). Taken together, these data show *S. aureus* and *L. monocytogenes* strains exposed to a non-lethal dose of tunicamycin exhibited moderate levels of resistance to ampicillin, tetracycline, meropenem, and erythromycin compared to the unexposed control bacterial cells, except for tetracycline in *L. monocytogenes*, where no cross-resistance was observed (**Figure 3**).

### Tunicamycin at Subinhibitory Concentrations Affects WTA Biosynthesis, Cell Morphology, and Colony Scatter Signatures

**FIGURE 4:**
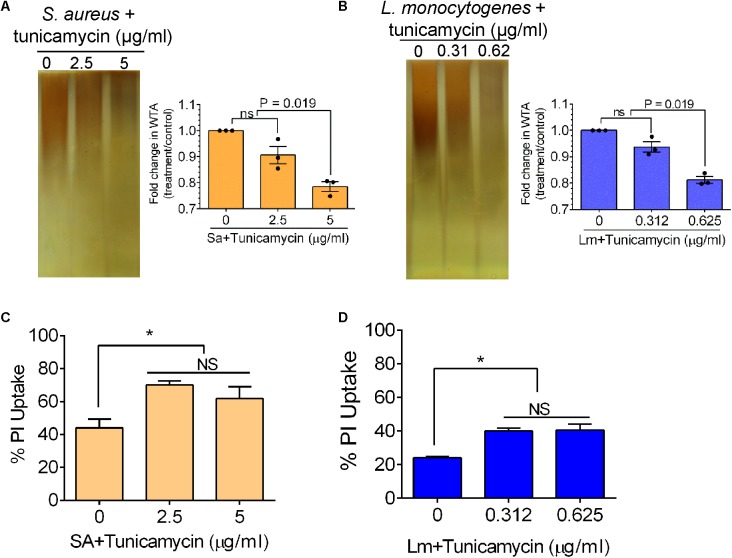
Analysis of wall teichoic acid (WTA) expression, and cell membrane permeability of *S. aureus* and *L. monocytogenes* after growth in the presence of subinhibitory concentrations of tunicamycin (2.5 – 5 μg/ml for *S. aureus*; 0.31 – 0.62 μg/ml for *L. monocytogenes*) for 24 h. Non-denaturing polyacrylamide gel electrophoresis (PAGE) followed by Alcian blue and silver staining of WTA preparation and quantitative estimation of WTA in *S. aureus* **(A)** and *L. monocytogenes* **(B)**. WTA was extracted from *S. aureus* (10^8^ CFU/ml) and *L. monocytogenes* (10^9^ CFU/ml). Percent propidium iodide (PI) uptake by tunicamycin-pretreated *S. aureus* **(C)** and *L. monocytogenes* **(D)** cells indicating increased cell wall/membrane permeability due to tunicamycin treatment. ^∗^*P* < 0.05; ns, no significance.

**FIGURE 5:**
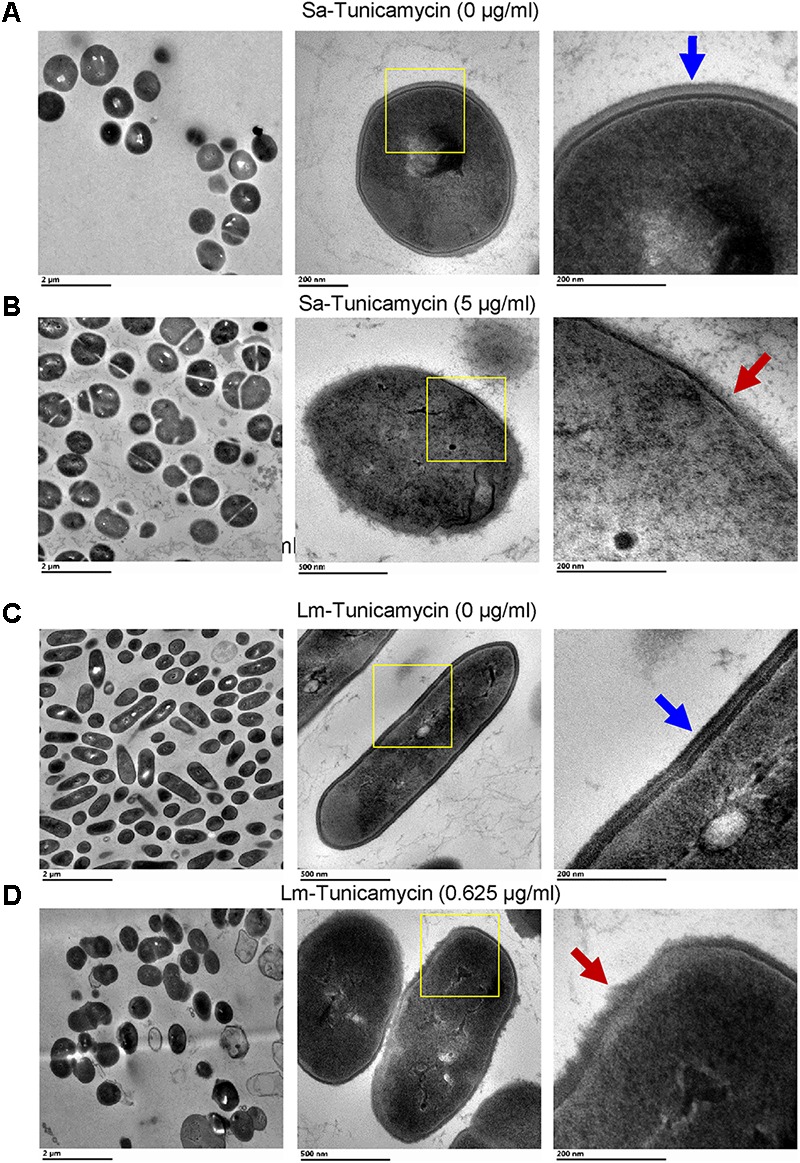
Transmission electron microscopy (TEM) images of *S. aureus* (Sa) **(A,B)** and *L. monocytogenes* (Lm) cells **(C,D)** after growth in the presence of subinhibitory concentrations of tunicamycin (5 μg/ml for *S. aureus*; 0.625 μg/ml for *L. monocytogenes*) for 24 h. Tunicamycin pretreatment caused an alteration in cell shape, cell division, morphology and cell wall ultrastructure of both *S. aureus* (**B**, red arrow) and *L. monocytogenes* (**D**, red arrows). Cell wall structure remained intact in untreated control cells (**A,C**, blue arrows). Boxed areas in the middle panel have been magnified and presented in the right panel.

To analyze the effect of tunicamycin at the subinhibitory concentrations on the WTA biosynthesis of *S. aureus* (2.5 and 5 μg/ml) and *L. monocytogenes* (0.312 and 0.625 μg/ml), non-denaturing-PAGE was performed. The WTA extracts were prepared from approximately the same number of bacterial cells by adjusting absorbance (A_600nm_) to 1, and Alcian blue and silver staining of PAGE revealed that the total amount of WTA was reduced by 18-20% in tunicamycin-treated *S. aureus* (**Figure 4A**) and *L. monocytogenes* (**Figure 4B**) cell wall fractions compared to the control (untreated) cells. These data also suggest tunicamycin–mediated inhibition of WTA biosynthesis is dose-dependent in both pathogens. Furthermore, percent propidium iodide (PI) uptake data revealed a significantly (*P* < 0.05) increased cell membrane permeability in both *S. aureus* and *L. monocytogenes* pre-treated with tunicamycin compared to the untreated controls (**Figures 4C,D**) indicating destabilized cell wall/membrane structure (Novo et al., 2000). Transmission electron microscopy (TEM) confirmed, alteration in cell shape, morphology and damage in the cell wall ultrastructure with disintegrated peptidoglycan architecture for both *S. aureus* (**Figure 5B**) and *L. monocytogenes* (**Figure 5D**) pre-treated with tunicamycin at 5 μg/ml and 0.625 μg/ml, respectively. While cell morphology and the cell wall ultrastructure remained intact in untreated control cells (**Figures 5A,C**). Tunicamycin-treated *S. aureus* cells showed incomplete cell division (**Figure 5B**, left panel), while rod-shaped *L. monocytogenes* cells appeared spherical (**Figure 5D**, left panel).

**FIGURE 6:**
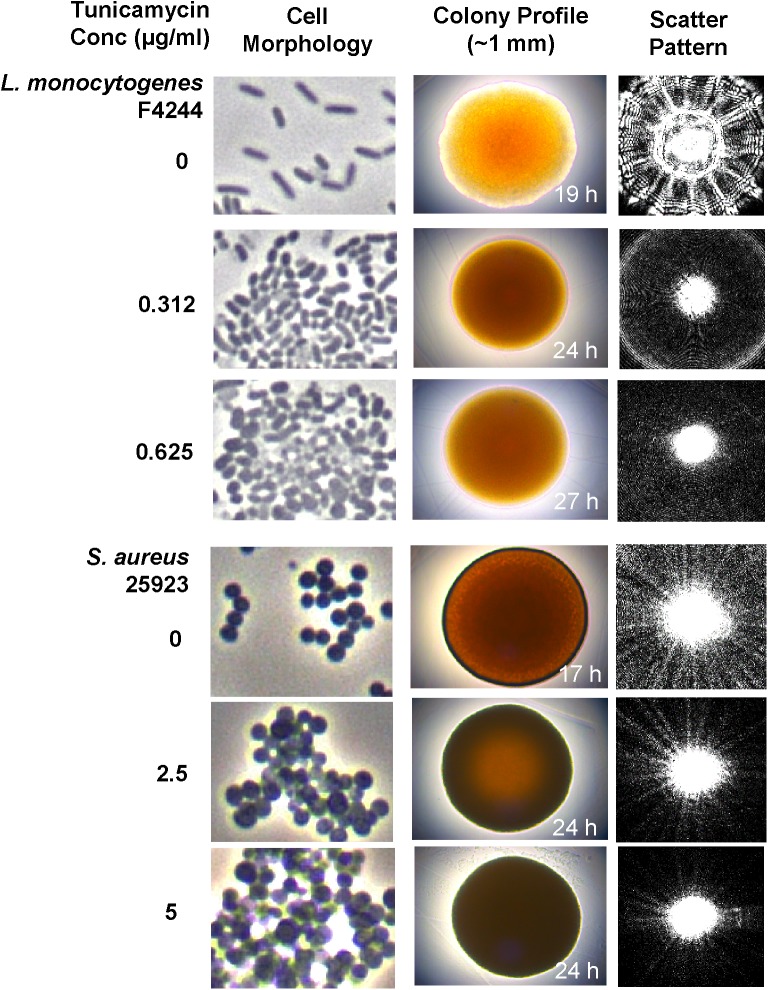
Analysis of cell morphology, colony profile, and colony scatter patterns of *S. aureus* and *L. monocytogenes* after growth in the presence of subinhibitory concentrations of tunicamycin (0, 2.5, and 5 μg/ml for *S. aureus*; 0, 0.31 and 0.625 μg/ml for *L. monocytogenes*) for 24 h. Phase-contrast microscopic imaging of live bacterial cell morphology (1000x), colony profile and corresponding light scatter signature of colonies of *L. monocytogenes* and *S. aureus* grown on TSA containing subinhibitory concentrations of tunicamycin.

Examination of single cell morphology and appearance under phase contrast microscopy revealed the formation of aggregates and clumping for both *L. monocytogenes* and *S. aureus* cells with increasing concentration of tunicamycin (**Figure 6** and **Supplementary Figure S1**). *L. monocytogenes* cells also appeared spherical. Colony morphology and the laser-induced scatter patterns (Singh et al., 2015) of bacteria grown in the presence of tunicamycin on TSA also revealed a substantial difference in colony appearance and subsequent scatter patterns (**Figure 6**). Tunicamycin treatment exerted the highest effect on the colony appearance and scatter patterns of *L. monocytogenes* than *S. aureus*. *L. monocytogenes* colony grown in the absence of tunicamycin appeared translucent with a slightly irregular wavy edge, while in the presence of tunicamycin the colonies appeared opaque, circular with smooth edges. The corresponding scatter patterns also showed contrasting differences. In the absence of tunicamycin (control), *L. monocytogenes* colony produced a typical scatter patterns with radial spokes while in the presence of antibiotics, the scatter patterns exhibited very faint regularly spaced concentric rings without the typical radial spokes (**Figure 6** and **Supplementary Figure S2**). In *S. aureus*, however, overall, the colonies appeared opaque and the changes were subtle (**Figure 6** and **Supplementary Figure S3**). The radial spokes were visible in the colony scatter patterns in the absence of antibiotic (control) while with increasing amounts of antibiotic, radial features of the scatter pattern were diminished (**Supplementary Figure S3**). Low-resolution in *S. aureus* scatter pattern can be attributed to the opaque colony, which interferes with laser propagation. Nevertheless, these results indicate that the interference in WTA biosynthesis exerts a considerable effect on individual cell structure and morphology, colony profile and subsequent optical scatter patterns (Banada et al., 2009; Singh et al., 2015).

### Tunicamycin Reduces Bacterial Ability to Form Biofilm

**FIGURE 7:**
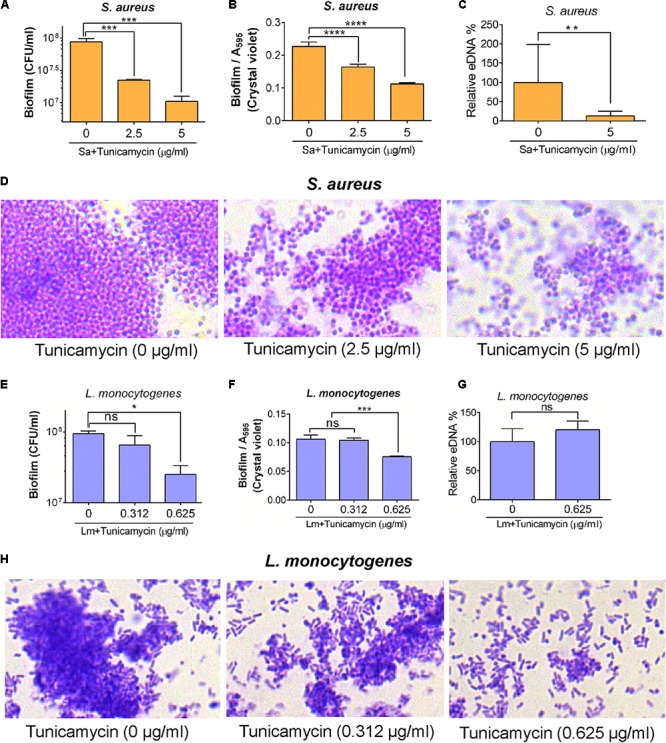
Biofilm formation and eDNA (extracellular DNA) release by *S. aureus* ATCC 25923 **(A–D)** and *L. monocytogenes* F4244 **(E–H)** pre-exposed to subinhibitory concentrations of tunicamycin for 24 h. Biofilm analysis by enumerating cell counts **(A,E)**, crystal violet dye uptake **(B,F)**, eDNA release **(C,G)** and light microscopic imaging of Gram-stained biofilms **(D,H)** after 48 h. Three independent experiments were performed to test biofilm formation and errors were represented as the standard error of the mean (SEM). ^∗∗∗∗^*P* < 0.0001, ^∗∗∗^*P* < 0.001; ^∗∗^*P* < 0.01; ^∗^*P* < 0.05; ns, no significance.

To analyze whether tunicamycin can influence the biofilm-formation ability of *S. aureus* 25923 and *L. monocytogenes* F4244, the bacterial cells were exposed to the subinhibitory concentration of tunicamycin for 24 h, and biofilm-formation on glass or plastics was assessed by both quantitative and qualitative assays after 48 h. Tunicamycin pre-exposure at both 2.5 and 5 μg/ml significantly (*P* < 0.05) lowered the biofilm-forming ability of *S. aureus* strain showing reduced bacterial counts in the biofilm (**Figure 7A**), crystal violet uptake (**Figure 7B**), and extracellular DNA (eDNA) level (**Figure 7C**). Furthermore, microscopic analysis of Gram-stained biofilms formed on glass slides clearly showed a fewer, and smaller biofilm clusters in the presence of increasing concentration of tunicamycin while the tunicamycin-untreated control cells produced much larger multilayered dense biofilm clusters (**Figure 7D**). A similar trend was seen in *L. monocytogenes* F4244 strain, however, tunicamycin only at the highest concentration (0.625 μg/ml) interfered with the biofilm formation compared to the control (no tunicamycin) and tunicamycin level at 0.312 μg/ml (**Figures 7E–H**). We did not observe tunicamycin effect on eDNA release from *L. monocytogenes* biofilm (**Figure 7G**). Collectively, these results clearly illustrate that tunicamycin–mediated interference with WTA biosynthesis reduced the biofilm-formation ability of both *S. aureus* and *L. monocytogenes*.

### Tunicamycin Treatment Reduces Bacterial Ability to Adhere and Invade Enterocyte-Like Caco-2 Cells

**FIGURE 8:**
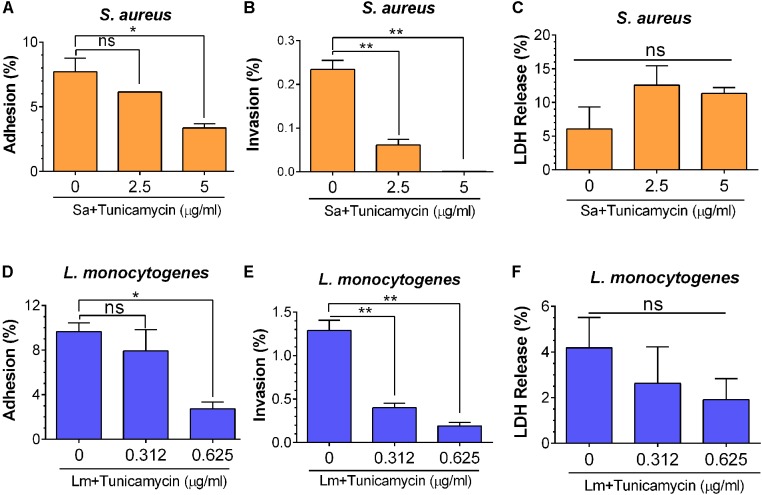
Bacterial adhesion **(A,D)**, invasion **(B,E)**, and lactate dehydrogenase (LDH) release **(C,F)** characteristics of *S. aureus* ATCC 25923 **(A–C)** and *L. monocytogenes* F4244 **(D–F)** of tunicamycin pre-exposed cells for 24 h from Caco-2 cells (MOI 10). LDH release was analyzed from the Caco-2 cells to ensure mammalian cell viability during exposure to bacteria. Data are average of three independent experiments ± SEM. ^∗∗^*P* < 0.01; ^∗^*P* < 0.05; ns, no significance.

To decipher the role of tunicamycin in cellular functional attributes in virulence, such as adhesion and invasion, *S. aureus* and *L. monocytogenes* were grown in the presence of subinhibitory concentrations of tunicamycin for 24 h and assayed for adhesion to, and invasion into the Caco-2 cells (Burkholder and Bhunia, 2010). *S. aureus* adhesion was significantly (*P* = 0.0364) reduced (3.4%) when the bacterium was cultured in the presence of 5 μg/ml of tunicamycin compared to the control cells (7.7% adhesion), while 2.5 μg/ml of tunicamycin treatment did not cause any significant difference compared to the control cells (**Figure 8A**). Likewise, invasion of *S. aureus* (treated with 2.5 μg/ml or 5 μg/ml of tunicamycin) was significantly (*P* = 0.0027) reduced compared to the untreated control cells (0.23%) (**Figure 8B**). Even though *S. aureus* is not an intracellular pathogen, but it can be internalized by epithelial or phagocytic cells, albeit at very low levels.

Similarly, adhesion of *L. monocytogenes* was significantly (*P* = 0.049) reduced from 9.7 to 2.7% when bacteria were pre-treated with 0.625 μg/ml tunicamycin (**Figure 8D**) and no significant difference was observed when the bacteria were treated with a lower concentration of tunicamycin (0.312 μg/ml). However, *L. monocytogenes* invasion was significantly (*P* = 0.0041) impaired when bacteria were pretreated with tunicamycin at 0.312 μg/ml or 0.625 μg/ml compared to the control cells (**Figure 8E**). However, when *S. aureus* or *L. monocytogenes* were exposed to Caco-2 cells simultaneously with the subinhibitory concentrations of tunicamycin for 1 h, we did not observe any difference in bacterial adhesion (data not shown). This suggests a 1-h exposure to antibiotics may not be sufficient to cause cell wall damage to interfere with bacterial adhesion. LDH release analysis from Caco-2 cells during bacterial adhesion and invasion experiments indicated that the Caco-2 cells remained viable without any significant changes in LDH values suggesting that the antibiotic-induced lower adhesion/invasion in both pathogens was due to impaired WTA biosynthesis and not due to Caco-2 cell damage (**Figures 8C,F**). Collectively, these results illustrate that WTA in both *S. aureus* and *L. monocytogenes* plays an important role in bacterial adhesion and invasion possibly by interfering with cell wall structural components that support the stable expression of adhesion or invasion associated virulence proteins (see below).

### Tunicamycin Affects Expression of Key Virulence Proteins

**FIGURE 9:**
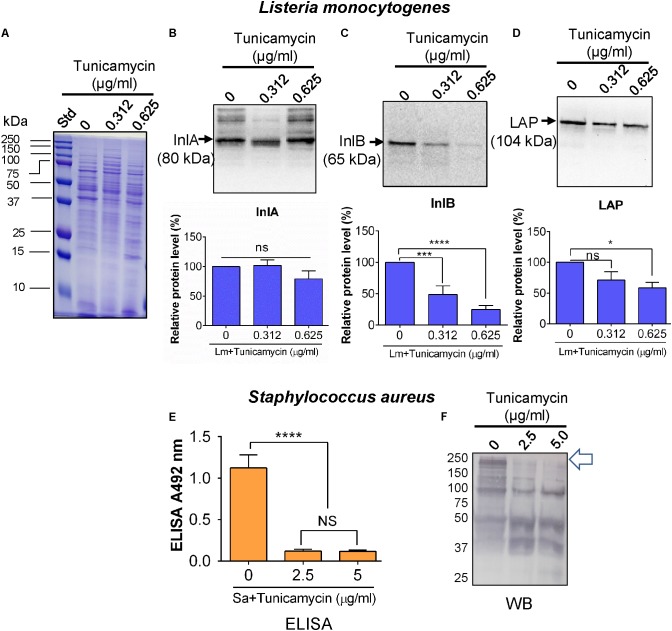
Immuno-analysis of expression of cell wall-associated surface proteins in *L. monocytogenes* (Lm) F4244 **(A–D)** and *S. aureus* ATCC 25923 **(E,F)** after pre-exposure to tunicamycin for 24 h. **(A)** Coomassie blue stained SDS-PAGE showing protein loading for *L. monocytogenes*. Immunoblots showing a reaction with anti-Listeria antibodies specific for Internalin A (InlA) **(B)**, Internalin B (InlB) **(C)** and *Listeria* adhesion protein (LAP) **(D)** and their corresponding quantitative data obtained using an image analysis software, ImageJ (NIH, Bethesda, MD, United States). **(E)** ELISA and **(F)** Western blot assay showing expression of SasA protein and cell wall proteins, respectively in *S. aureus* after exposure to tunicamycin at 2.5 μg/ml and 5.0 μg/ml for 24 h. Arrow points to the expression of high molecular weight proteins in tunicamycin untreated *S. aureus* cells, which were diminished in tunicamycin-treated cells. ^∗∗∗∗^*P* < 0.0001; ^∗∗∗^*P* < 0.001; ^∗^*P* < 0.05; ns, no significance.

To investigate the influence of tunicamycin on expression of cell wall-associated key adhesion and invasion proteins, InlA (Gaillard et al., 1991), InlB (Braun et al., 1997), and LAP (Jagadeesan et al., 2010) in *L. monocytogenes*, and on SasA, a major MSCRAMM (Rivas et al., 2004; Otto, 2008; Yang et al., 2016) in *S. aureus*, immunoassays were performed. Both pathogens were cultured in the presence of a subinhibitory concentration of tunicamycin for 24 h; proteins were extracted from the cell wall fractions and immunoprobed with appropriate antibodies. Equal loading of proteins was ascertained with the Coomassie brilliant blue R-250 staining (**Figure 9A**). The relative protein levels of InlB (**Figure 9C**) and LAP (**Figure 9D**) in *L. monocytogenes* F4244 treated with tunicamycin (0.312 and 0.625 μg/ml) were significantly (*P* < 0.0001) reduced to 48.8 and 24.8% for InlB and 41.3% for LAP, respectively, compared to the respective antibiotic untreated controls. There was no significant difference of relative InlA-expression in the presence or absence of tunicamycin (**Figure 9B**).

For *S. aureus*, we used a mAb in ELISA against SasA (Yang et al., 2016), a major protein component of MASCRMM to assess the expression level of this protein after tunicamycin exposure. The anti-SasA mAb showed a significantly higher reaction (*P* < 0.05) with untreated *S. aureus* cells compared to tunicamycin (2.5 and 5 μg/ml)-treated cells (**Figure 9E**). This indicates that tunicamycin treatment interfered with the expression of SasA protein, which is a part of MSCRAMM. We also examined overall cell wall protein expression levels using a commercial anti-*S. aureus* pAb in Western blot and data show reduced expression of high molecular weight cell surface proteins in tunicamycin-treated cells (**Figure 9F**).

### Tunicamycin Pre-exposure Attenuates Bacterial Inflammatory Response

**FIGURE 10:**
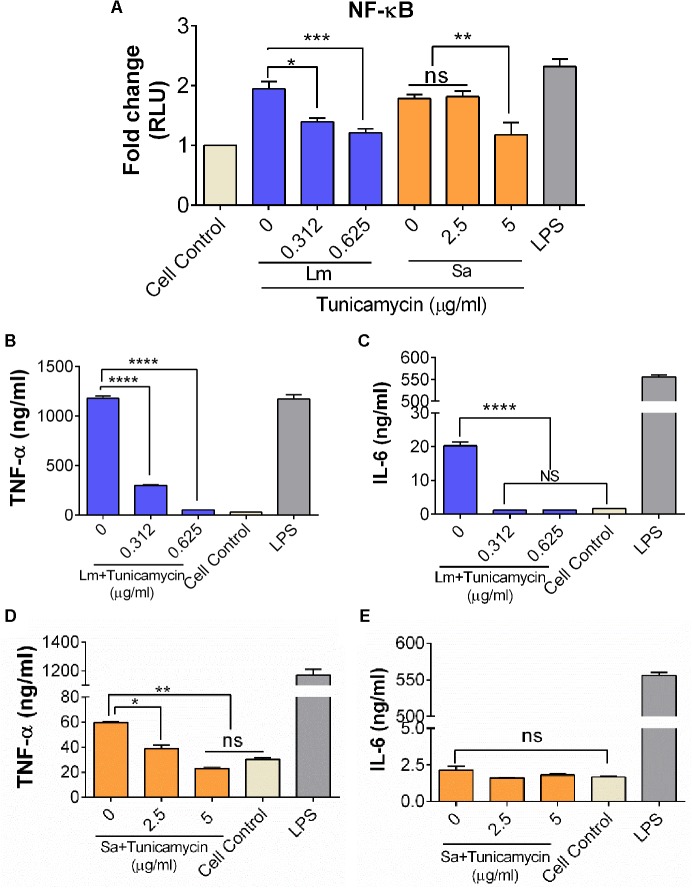
Analysis of NF-κB activation **(A)** and inflammatory cytokine production **(B–E)** from murine macrophage cell line, RAW 264.7. **(A)** Luciferase NF-κB/LUCPorter^TM^ reporter RAW 264.7 cell line assay to monitor expression of NF-κB, after 6 h exposure to *S. aureus* (Sa) and *L. monocytogenes* (Lm) cells that were pre-exposed to tunicamycin for 24 h. TNFα and IL-6 release by *L. monocytogenes* **(B,C)**, and *S. aureus* **(D,E)**. Cell control, cells without any treatment was used as a background control and LPS (lipopolysaccharide) from *E. coli* was used as a positive control in each experiment. Data are average of two independent experiments performed in triplicate ± SEM (*n* = 6). ^∗∗∗∗^*P* < 0.0001; ^∗∗∗^*P* < 0.001; ^∗∗^*P* < 0.01; ^∗^*P* < 0.05; ns, no significance.

To assess the effect of WTA-inhibiting tunicamycin on the inflammatory response of *S. aureus* and *L. monocytogenes*, we used a murine macrophage luciferase-reporter cell line (RAW 264.7) to assay the level of NF-κB expression after 6 h exposure to bacteria (**Figure 10A**). Relative to control uninfected cells, *L. monocytogenes* F4244 without tunicamycin treatment caused 1.94-fold higher expression of NF-κB while tunicamycin (0.312 μg/ml and 0.625 μg/ml) pretreated *L. monocytogenes* (24 h) showed 1.4-fold and 1.2-fold higher expression, respectively. Likewise, relative to control uninfected cells, *S. aureus* without tunicamycin treatment (24 h) caused 1.8-fold higher NF-κB expression while tunicamycin-treated *S. aureus* at 5 μg/ml, induced 1.2-fold higher expression (*P* < 0.0001) (**Figure 10A**). *Escherichia coli* derived lipopolysaccharide (LPS) was used as a positive control, which showed a significantly (*P* < 0.05) higher expression than the control cells (**Figure 10A**).

The effect of a crude WTA preparation from tunicamycin pre-treated bacterial cells on inflammatory cytokines (TNFα, IL-1β, and IL-6) production from RAW 264.7 macrophage cells was assayed. WTA preparation from control (tunicamycin-untreated) *L. monocytogenes* cells showed significantly higher levels of TNFα and IL-6 compared to the tunicamycin treatment at two concentrations (0.312 μg/ml and 0.625 μg/ml) (**Figure 10B**). Likewise, WTA from *S. aureus* cells showed a significantly higher level of TNFα (*P* < 0.05) compared to the tunicamycin treatment at two concentrations (2.5 and 5 μg/ml); however, no difference in IL-6 production was observed (**Figure 10E**). LPS as a positive control induced high levels of both TNFα and IL-6 (**Figures 10B,C**). IL-1β was undetectable in RAW 264.7 cell supernatants under all treatment conditions (data not shown). Altogether, these data demonstrate that WTA in both *L. monocytogenes* and *S. aureus* are responsible for strong inflammatory response, which could be attenuated by the tunicamycin-mediated inhibition of WTA biosynthesis.

## Discussion

In pathogenic Gram-positive bacteria, the cell wall plays an important role not only in bacterial physiology but also in pathogenesis and host defense immune response including complement activation, phagocytosis (opsonization) and T-cell activation (Weidenmaier and Peschel, 2008; Kurokawa et al., 2016). The cell wall is comprised of peptidoglycan, teichoic acids, teichuronic acid, lipoglycan, and polysaccharide. Cell wall also serves as a scaffold for anchoring of many adhesion and invasion proteins. Teichoic acid, also known as WTA is an anionic glycopolymer, which is covalently attached to the peptidoglycan and extends outwardly (Brown et al., 2013). WTA constitutes up to 60% of the cell wall found exclusively in the Gram-positive bacteria and plays important role in bacterial physiology, cell morphology and cell division, autocatalytic activity, ion homeostasis, and pathogenesis (Ellwood, 1970; Weidenmaier and Peschel, 2008; Swoboda et al., 2010).

In addition, tunicamycin inhibits *N*-acetylglucosamine-1-phosphate transferase such as TarO and MraY that catalyze an early stage reaction in peptidoglycan cell wall assembly (Campbell et al., 2011). TarO is the first enzyme in the WTA biosynthetic pathway, while MraY is the secondary target in peptidoglycan pathway (Watkinson et al., 1971; Price and Tsvetanova, 2007; Campbell et al., 2011; Hakulinen et al., 2017). Tunicamycin is one of the antibiotics that affect the WTA biosynthesis (Swoboda et al., 2009) and it has been used against methicillin-resistant *S. aureus* (MRSA) and to sensitize against beta-lactam antibiotics (Campbell et al., 2011), where beta-lactam antibiotics alone is less effective on MRSA (Guignard et al., 2005).

In this study, we show that WTA inhibiting antibiotic, tunicamycin at the subinhibitory dosage affects *S. aureus* and *L. monocytogenes* cell morphology, cell division, cell wall ultrastructure, biofilm formation and pathogenesis, but promotes moderate antibiotic cross-resistance to select therapeutic antibiotics. Accomplishing WTA inhibition by using tunicamycin without affecting the viability of *S. aureus* and *L. monocytogenes* was essential in understanding the role of WTA in antibiotic resistance and pathogenesis. MIC has been interpreted as a gold standard to evaluate the resistance of bacteria to antimicrobials. Depending on the pathogen, the MIC values vary, but here, we observed growth media dependent variation in MIC values for a pathogen (**Figures 1, 2**). For example, the MIC of tunicamycin for *S. aureus* strain in TSB was 20 – 40 μg/ml while >40 μg/ml in TSBYE and MHB and a similar high trend was observed for *L. monocytogenes*. This could be attributed to the difference in ionic strength, pH, aeration, and temperature of growth media, which affect bacterial susceptibility (Yeaman and Yount, 2003). Such difference in MIC values was also previously observed for antimicrobial peptides Cecropin A and Magainin 2 (Choi et al., 2014). Since both *S. aureus* and *L. monocytogenes* exhibited high sensitivity to the tunicamycin, TSB was chosen for further studies.

After establishing the MIC and subinhibitory concentrations of tunicamycin on *S. aureus* and *L. monocytogenes*, we examined the effect of tunicamycin on bacterial cross-resistance to therapeutic antibiotics such as erythromycin, gentamycin, vancomycin, rifampicin, ampicillin, tetracycline, and meropenem (a broad-spectrum carbapenem antibiotic) (**Figure 3**). Tunicamycin pre-exposed both pathogens showed an increase in the MIC values for erythromycin, ampicillin, and meropenem while *S. aureus* showed resistance to additional antibiotics, tetracycline. Cross-resistance among unrelated antibiotics with dissimilar chemical structure and mode of action is not fully understood (Sutherland et al., 1964); However, increased resistance may be attributed to increased uptake of these antibiotics by the tunicamycin pre-treated cells as these cells showed increased membrane permeability (**Figure 4**). It has been reported that bacteria cultured in the presence of any one of penicillin, chloramphenicol, tetracycline, or others, developed cross-resistance for another antibiotic (Szybalski and Bryson, 1952). Therefore, the acquired resistance in bacteria to a specific antibiotic may develop new properties simultaneously, including resistivity to other chemotherapeutic agents.

Antibiotic susceptibility of resistant strains may vary from lower, equal, or higher compared to the parent culture for an antibiotic that was not used in their isolation (Szybalski and Bryson, 1952). Due to a limited literature available on antimicrobial cross-resistance, it is utterly important to pursue this area of research to gain in-depth knowledge in the development of antimicrobial cross-resistance to therapeutic antibiotics. Contrary to our finding, previous studies have shown that inhibiting WTA render bacteria sensitive to β-lactam antibiotics (Brown et al., 2012). This can be argued as, (i) subinhibitory exposure of tunicamycin might have triggered a global adaptive response to the antibiotic stress (Fajardo and Martínez, 2008; Andersson and Hughes, 2014), and (ii) inhibition of WTA renders less negative charge on bacterial cell surface, which limits the ionic interactions between the antibiotics and the bacterial cell surface. This may explain the emergence of cross-resistance in *S. aureus* and *L. monocytogenes* pre-exposed to subinhibitory concentrations of tunicamycin. Inhibition of teichoic acid also affected cell membrane permeability as PI uptake was increased in tunicamycin-pretreated cells (**Figure 4**) (Novo et al., 2000; Elbaz and Ben-Yehuda, 2010). TEM images confirmed tunicamycin-induced incomplete cell division, damage in the cell wall ultrastructure, and alteration in cell morphology of both pathogens (**Figure 5**). Furthermore, the rod-shaped *L. monocytogenes* cells appeared spherical (**Figure 5**). A similar spherical structure was also observed for *Bacillus subtilis* with reduced WTA expression (Pollack and Neuhaus, 1994). The reduced net negative charge on the bacterial surface also caused bacteria to form aggregates as was seen in the light microscopic images (**Figure 6**). Furthermore, tunicamycin-mediated reduced WTA level in individual cells in a colony also affected bacterial colony profile from translucent to dense opaque appearance and subsequent changes in optical scatter patterns indicating that cell wall composition can severely influence bacterial colony scatter signatures (Banada et al., 2009). Previously we observed that subinhibitory concentration of streptomycin significantly altered *Salmonella enterica* serovar Typhimurium colony scatter patterns due to the production of the increased amount of stress response protein, GroEL (Singh et al., 2015).

Wall teichoic acid from *S. aureus* is also reported to play important role in adhesion of bacteria and in biofilm-formation (Gross et al., 2001; Vergara-Irigaray et al., 2008; Swoboda et al., 2010). Interestingly, we observed that the biofilm formation ability of both *S. aureus* and *L. monocytogenes* was significantly reduced when these cells were treated with different amounts of WTA-inhibiting tunicamycin (**Figure 7**). This is in agreement with a previous report where authors indicated that disturbance in bacterial cell wall structure affect bacterial ability to form biofilm (Bucher et al., 2015). Consequently, eDNA release was also significantly decreased in tunicamycin pretreated *S. aureus* cells than the untreated control cells (Mann et al., 2009) indicating reduced eDNA directly correlates with lowered biofilm formation; however, no change was observed for *L. monocytogenes* in spite of reduced biofilm formation. This later event indicates eDNA release and its contribution to biofilm formation may vary among bacterial genera (Montanaro et al., 2011).

Tunicamycin treatment also reduced bacterial adhesion to and invasion in Caco-2 cell line (**Figure 8**), which could be enabled by two distinct mechanisms, (i) direct and (ii) indirect. In the direct mechanism, WTA, an amphiphilic molecule directly helps bacterial adhesion to biological surfaces. In this study, tunicamycin treatment (5 μg/mI) of *S. aureus* significantly reduced its ability to adhere or invade Caco-2 cells possibly due to inhibition of WTA biosynthesis. In a previous study, pre-incubation of nasal epithelial cells with WTA, reduced adhesion of *S. aureus* by 71% (Aly et al., 1980). In another study, colonization of WTA-free *S. aureus* to rat nares reduced by 90% compared to those of wild-type *S. aureus* (Weidenmaier et al., 2004). In addition, studies have shown that reduction in WTA synthesis in bacterial strains due to mutation or by using antibiotics were able to significantly inhibit bacterial ability to colonize and infect animals (Weidenmaier et al., 2004; 2005). Suzuki et al (Suzuki et al., 2011) showed that WTA can promote *S. aureus* invasion of corneal epithelial cells.

In case of *L. monocytogenes*, tunicamycin treatment also significantly reduced adhesion to Caco-2 cells. Attachment of D-alanine esters to WTA, called D-alanylation is an important mechanism that Gram-positive bacteria use for the modulation of surface charge. Abachin et al. (2002) showed that alanylation of teichoic acid is required for adhesion and virulence of *L. monocytogenes*. In *Streptococcus pyogenes*, mutations in genes responsible for teichoic acid D-alanylation resulted in diminished adhesion and invasion of cultured human pharyngeal epithelial cells, and resistance to antimicrobial peptides (Kristian et al., 2005). In *Lactobacillus johnsonii*, teichoic acid helped bacterial adhesion to Caco-2 cells, whereas a teichoic acid deficient isogenic strain failed to adhere to Caco-2 cells (Granato et al., 1999). These provide strong evidence for the direct involvement of WTA in bacterial adhesion and invasion to mammalian cells, which could be impaired by WTA-inhibiting tunicamycin. WTA contributes to cation homeostasis (Weidenmaier and Peschel, 2008), and serves as a scaffold for binding to other molecules including many surface-associated virulence factors, such as MSCRAMM in *S. aureus* (Clarke and Foster, 2006), and Internalin B (InlB) in *L. monocytogenes* (Braun et al., 1997; Jonquieres et al., 1999).

In the indirect mechanism, tunicamycin pre-exposure impaired WTA synthesis thus prevented anchoring or surface display of key adhesion/invasion proteins. This may result in reduced bacterial adhesion or invasion into mammalian cells. For example, in *L. monocytogenes*, InlA, InlB, and LAP play important role in *L. monocytogenes* adhesion and invasion (Camejo et al., 2011). Here, tunicamycin treatment lowered expression of InlB and LAP, but not the InlA (**Figure 8**). InlA uses a C-terminal cell wall anchoring domain (LPXTG motif) to covalently interact with the peptidoglycan (Bierne and Cossart, 2007) and its level was not affected by tunicamycin treatment indicating that InlA-mediated adhesion or invasion to Caco-2 cells was not likely impaired by WTA biosynthesis. InlB, another adhesion and invasion protein of Internalin-multigene family (Bierne and Cossart, 2007), on the other hand, possess GW (glycine-tryptophan) module to anchor with WTA through noncovalent interaction (Jonquieres et al., 1999; Bierne and Cossart, 2007). Tunicamycin-mediated inhibition of WTA biosynthesis justifiably affected InlB surface expression and subsequent Caco-2 invasion (**Figures 8, 9**). LAP is a secreted protein and it is postulated to re-associate on the surface of the bacterium after secretion (Burkholder et al., 2009; Jagadeesan et al., 2010). Here, LAP expression was significantly reduced in tunicamycin-treated cells, suggesting that WTA in the cell wall may be an important molecule that possibly interacts with LAP. In *S. aureus*, the major adhesion protein complex is MSCRAMM, which interacts with WTA in the cell wall. SasA, the major MSCRAMM protein was significantly reduced due to tunicamycin treatment (Yang et al., 2016) thus may have resulted in reduced *S. aureus* adhesion/invasion to Caco-2 cells. Kukita et al. (2013) reported that SasA also binds to the salivary agglutinin gp340 protein derived from human saliva. These findings demonstrate that tunicamycin-mediated inhibition of WTA can interfere with pathogens’ ability to adhere and invade host cells.

Wall teichoic acid also induces inflammation due to activation of NF-κB through TLR-2 mediated pathway (Matsuguchi et al., 2003; Schröder et al., 2003). Here tunicamycin treated *L. monocytogene*s and *S. aureus* cells showed reduced NF-κB activity and lower levels of inflammatory cytokines, TNFα and IL-6 (**Figure 10**) suggesting that inhibition of WTA biosynthesis or WTA anchored virulence proteins (InlB and LAP in *L. monocytogenes*; and SasA in *S. aureus*) was possibly responsible for such reduced inflammatory response (Mansell et al., 2000; Drolia et al., 2018).

In summary, our results indicate that exposure of Gram-positive bacterial pathogens to non-lethal (subinhibitory) dose of tunicamycin inhibits WTA biosynthesis thus affecting cell morphology, cell division, cell wall ultrastructure, biofilm formation, and bacterial pathogenesis (adhesion and invasion potential) and host immune response. Furthermore, inhibition of WTA biosynthesis also increases cell membrane permeability hence increased uptake of antibiotics and development of cross-resistance to several therapeutic antibiotics. The results from this study could help in better understanding the roles of WTA in bacterial pathogenesis and antibiotic resistance, which will help investigators in designing antimicrobials with enhanced therapeutic potential against Gram-positive bacterial pathogens.

## Materials and Methods

### Bacterial Strains and Growth Media

*Staphylococcus aureus* strains, ATCC 29213, ATCC 43300, ATCC 25923, and PRI 4656; and *Listeria monocytogenes* strains F4244, ATCC 43257, ATCC 15313, and 10403S were used in this study. Bacterial cultures were procured from either ATCC (American Type Culture Collection, Manassas, VA, United States) or Presque Isle (Erie, PA, United States). All bacterial cultures were maintained as 25% frozen glycerol stocks at −80°C and for fresh cultures, bacteria were grown in TSB with 0.6% yeast extract (TSBYE), tryptic soy broth (TSB, BD) or Muller Hinton broth (MHB) at 37°C at 130 rpm in an orbital shaker. All dehydrated media (Beckton Dickinson) were prepared as per the manufacturer’s instruction.

### Minimal Inhibitory Concentration and Subinhibitory Concentration Determination of Tunicamycin

Tunicamycin was purchased as a dehydrated powder (Tocris^TM^ Bioscience, Bristol, United Kingdom) and 10 mg/ml stocks were prepared in dimethyl sulfoxide (DMSO) solvent. The MIC of tunicamycin for *S. aureus* and *L. monocytogenes* cultures were determined by using a microtiter plate dilution method (Andrews, 2001). Briefly, the overnight cultures (16–18 h) of *S. aureus* and *L. monocytogenes* strains were incubated in TSBYE at 37°C at 130 rpm in an orbital shaker for 16–18 h. Bacterial cells were harvested by centrifugation (8000 ×*g*, 4 min); washed with PBS twice and resuspended in TSB to a final inoculum concentration of 10^6^ CFU/ml. An aliquot of 100 μL (10^5^ CFU) was added in duplicate to each well of a 96-well microtiter plate. Working concentrations of tunicamycin were also prepared in TSB and two-fold serially diluted in a microtiter plate to achieve a final concentration in the range of 0.156 μg/ml to 80 μg/ml for *S. aureus* and 0.039 μg/ml to 20 μg/ml for *L. monocytogenes*. Microtiter plate with samples was incubated at 37°C (with shaking at 70 rpm) for 24 h, and the absorbance at 600 nm was measured using a plate reader (BioTek Teknova, Hollister, CA, United States). Similarly, the MIC of bacterial cells also was repeated using MHB and TSBYE media. Tunicamycin was dissolved in TSBYE in the range of 0.156 - 80 μg/ml for *S. aureus*, and 0.039 – 20 μg/ml for *L. monocytogenes*, and in MHB in the range of 0.156 – 80 μg/ml for *S. aureus* and 0.009 – 5 μg/ml for *L. monocytogenes.*

To determine subinhibitory dose, bacterial growth-curve was generated in the presence of different concentrations of antibiotics using two strains of each *S. aureus* (strains ATCC 29213, ATCC 43300), and *L. monocytogenes* (strains F4244, ATCC43257). Bacteria were centrifuged (8000xg, 4 min), washed with PBS twice. The *S. aureus* and *L. monocytogenes* cultures were resuspended in TSB to a final concentration of 2 × 10^5^ CFU/ml and 1 × 10^6^ CFU/ml, respectively. An aliquot of 100 μl of bacteria was added to each respective well of a microtiter plate. To calculate the inhibitory effect of tunicamycin on *S. aureus*, tunicamycin was added per well and twofold serially diluted in TSB at 0 h time. The *S. aureus* strains were diluted to 1 × 10^5^ CFU/ml and *L. monocytogenes* strains were diluted to 5 × 10^5^ CFU/ml per well after adding the tunicamycin in 96 well templates. In *S. aureus* strains, tunicamycin was added ranging from 40 to 0.078 μg/ml. Similarly, *L. monocytogenes* were exposed to different concentration of tunicamycin ranging from 20 to 0.039 μg/ml. The plates were incubated at 37°C on an orbital shaker (70 rpm), and the optical density of each well was measured every hour. Three independent experiments were performed and data expressed as mean average value ± SEM. The subinhibitory concentration of tunicamycin for *S. aureus* was determined to be 2.5 μg/ml to 5 μg/ml and for *L. monocytogenes* 0.312 μg/ml to 0.625 μg/ml. Hence, in all subsequent experiments, *S. aureus* was cultured in TSB containing tunicamycin (2.5 μg/ml and/or 5 μg/ml) and *L. monocytogenes* in TSB with tunicamycin (0.312 μg/ml and/or 0.625 μg/ml) at 37°C for 24 h.

### Extraction of WTA and PAGE

Wall teichoic acid was extracted from each 40 mL bacterial culture as described before (Meredith et al., 2008). Bacterial cells (OD_600_ adjusted to 1) were harvested and washed once with 30 ml of Buffer 1 [50 mM 2-(*N*-morpholino) ethanesulfonic acid (MES), pH 6.5] and then resuspended in 30 ml of Buffer 2 [4% (w/v) sodium dodecyl sulfate (SDS), 50 mM MES; pH 6.5]. Samples were boiled for 1 h and then the cells were collected by centrifugation (10,000 ×*g*, 10 min). The cell pellets were resuspended in Buffer 2 and centrifuged (14,000 ×*g*, 10 min). The pellet was washed once with Buffer 2, once with Buffer 3 [2% NaCl, 50 mM MES, pH 6.5] and finally with Buffer 1. Samples were treated with proteinase K buffer [20 mM Tris-HCl (pH 8.0), 0.5% (w/v) SDS, and 20 μg of proteinase K in 1 ml] and incubated at 50°C for ∼4 h. Samples were washed once with buffer 3 and then three times with distilled water to remove the SDS. Samples were thoroughly resuspended in 1 ml of 0.1 M NaOH and shaken at room temperature for 16 h to hydrolyze WTA. Insoluble cell wall debris was removed by centrifugation (14,000 ×*g*, 10 min). The supernatant containing the hydrolyzed WTA was either directly analyzed by non-denaturing PAGE followed by staining with Alcian blue and silver stain (Min and Cowman, 1986; Pollack and Neuhaus, 1994) or stored at 4°C or lyophilized.

### Bacterial Surface Protein Expression Analysis by Western Blot and ELISA

Cell wall protein from 5 ml of each freshly grown bacterial cultures was extracted and quantified by bicinchoninic acid assay (BCA assay, Thermo Fisher) as described before (Burkholder et al., 2009). Thirty microliters of each protein sample were electrophoresed in 10% SDS-PAGE (sodium dodecyl sulfate-polyacrylamide gel electrophoresis) for 1.5 h at 100 V. Then the proteins were transferred to hydrophobic PVDF membrane (Thermo-Fisher) and were blocked with blocking buffer (5% non-fat dry milk in 0.1 M Tris-buffered saline [TBS with 0.1% Tween-20; TBS-T]) for 1 h at room temperature and washed 3 times with TBS-T for 5 min each. Subsequently, the membranes were probed with 0.5–1 μg/ml of each anti-lnlB pAb, anti-lnlA mAb, and anti-LAP mAb overnight at 4°C with gentle shaking (All antibodies were from our laboratory). Similarly, membranes containing *S. aureus* surface proteins were probed with anti-*S. aureus* pAb (1:1000; cat # PA1-7246; Thermo-Fisher). Membranes were again incubated with either anti-mouse or anti-rabbit antibody conjugated horseradish peroxidase and developed with ECL (Cell Signaling Technology). The band was finally visualized with Gel Doc^TM^ XR+ Gel Documentation System (Bio-Rad) and analyzed by Quantity One software (Bio-Rad).

ELISA was performed to assay for SasA protein levels in tunicamycin exposed *S. aureus*. The microtiter plate wells (Immunolon 4X HBX) were sensitized with *S. aureus* cells (1 × 10^7^ CFU/ml) using PBS (0.1 M, pH 7.4), and incubated overnight at 4°C. The plates were washed with TBS-T three times and blocked with ELISPOT blocking buffer (eBioscience^TM^ ELISA/ELISPOT Diluent Cat# 00-4202-56, Thermo-Fisher). The wells were probed with anti-SasA mAb (1 μg/ml) (Yang et al., 2016) and developed using rabbit anti-mouse horseradish peroxidase-conjugated antibody and o-phenyl diamine and hydrogen peroxide (Jagadeesan et al., 2010). Note, anti-SasA mAb does not work well in Western blot hence it was used in ELISA (Yang et al., 2016).

### Bacterial Cell and Colony Morphology, Light Scatter Pattern Analysis and Membrane Permeability Assay

Bacterial cells were examined under the phase-contrast light microscope and were photographed using a Leica microscope. To analyze the colony morphology and the colony scatter patterns, bacteria without tunicamycin treatment were serially diluted and plated on TSA, while antibiotic pre-exposed bacteria were plated on TSA containing tunicamycin (0.312 μg/ml or 0.625 μg/ml for *Listeria*, and 2.5 μg/ml or 5 μg/ml, for *S. aureus*). Plates were incubated at 37°C for 17–27 h or until the colony diameter reached 1.1 ± 0.2 mm. Colony images were acquired under a light microscope (100x magnification) (Leica) and colony scatter images of each pathogen, at least 40 colonies were collected using an automated BARDOT (BActerial Rapid Detection using Optical scattering Technology) machine (Singh et al., 2014, 2015, 2016).

To determine tunicamycin effect on bacterial cell wall structure and membrane permeability, both *S. aureus* and *L. monocytogenes* after growth (37°C for 24 h) in the presence of tunicamycin were adjusted to OD_595_ = 1.0, harvested, concentrated threefold, and propidium iodide (PI) added to each culture (1 μg/ml) and incubated in the dark for 10 min. Heat-treated (95°C for 10 min) bacteria were used as positive controls. Samples were transferred to a 96-well microtiter plate and measured at 485 nm (excitation) and 620 nm (emission) using a spectrofluorometer (SpectraMax). To calculate the percent PI uptake (permeability), the fluorescence reading of each sample was divided by the fluorescence from their corresponding positive controls (Novo et al., 2000).

### Transmission Electron Microscopy

*Listeria monocytogenes* and *Staphylococcus aureus* cultures were grown in TSB-YE (0.6%) for 24 h with or without treatment of tunicamycin at a concentration of 0.625 μg/ml and 5 μg/ml, respectively. Bacterial samples were washed thoroughly in PBS (pH 7.4) and fixed in 2.5% glutaraldehyde in 0.1 M sodium cacodylate buffer, post-fixed in buffered 1% osmium tetroxide containing 0.8% potassium ferricyanide, and en bloc stained in 1% aqueous uranyl acetate. Samples were then dehydrated with a graded series of acetonitrile and embedded in resin. Thin sections (80 nm) were cut on a Leica EM UC6 ultramicrotome and stained with 2% uranyl acetate and lead citrate. Images were acquired on an FEI Tecnai 12 electron microscope operating at 80 kV.

### Effect of Tunicamycin on Antibiotic Cross-Resistance

The effect of subinhibitory concentration of tunicamycin pre-exposure to *S. aureus* (2.5 μg/ml) and *L. monocytogenes* (0.312 μg/ml) on bacterial resistance to several antibiotics was tested by microdilution method (Andrews, 2001). A stock solution of ampicillin (10 mg/ml) in water, tetracycline (5 mg/ml) in water, meropenem (1 mg/ml) in DMSO (dimethyl sulfoxide), erythromycin (10 mg/ml) in pure ethanol, gentamycin (50 mg/ml) in water, vancomycin (25 mg/ml) in water, and rifampicin (40 mg/ml) in pure methanol were prepared and filter sterilized using 0.2 μm syringe filter (Millipore) except erythromycin and rifampicin. To calculate the MIC, antibiotics were 2-fold serially diluted in microtiter plate in TSB containing either ampicillin (0.078–40 μg/ml), tetracycline (0.0048–2.5 μg/ml), meropenem (0.0024–1.25 μg/ml), gentamycin (0.39–12.50 μg/ml), erythromycin (0.39–12.50 μg/ml), vancomycin (0.78–25.00 μg/ml), and rifampicin (0.01–0.43 μg/ml). Thereafter, *S. aureus* ATCC 25923 and *L. monocytogenes* F4244 cultured in the absence (control) or presence of tunicamycin (0.312 μg/ml for *Listeria*, and 2.5 μg/ml for *S. aureus*) at 37°C for 24 h were diluted in TSB to achieve a concentration of 1 × 10^6^ CFU/ml. An inoculum of 100 μL (1 × 10^5^ CFU) was added in triplicate to each well of a 96-well plate containing twofold serially diluted antibiotics. Microtiter plates were then incubated at 37°C for 24 h on an orbital shaker (90 rpm), and absorbance was measured at 600 nm.

### Biofilm Assay and eDNA Analysis

Effect of subinhibitory concentration of tunicamycin on bacterial biofilm formation was analyzed by (i) plate counting, (ii) microtiter-based crystal violet staining (Djordjevic et al., 2002) and (iii) Gram-staining. Freshly grown each culture was adjusted to OD_595 nm_ = 1.4 and diluted (1:200) in TSB (47 ml) and the entire volume was added to a tissue culture treated Petri dishes (15 × 15 cm, TPP, Switzerland) and incubated at 30°C for 48 h to allow biofilm formation. Petri plates were rinsed with PBS (5 ml) twice to remove loosely bound bacteria and adhered cells were harvested in 5 ml PBS using sonication for 15 min (iSonic Inc, Chicago, IL, United States), and cell scraper was used to remove the bound cells, serially diluted and plated on TSBYE agar to enumerate biofilm-forming bacteria. Three independent experiments were performed and data expressed as mean average value ± SEM.

For microtiter plate based biofilm assay, bacterial samples were prepared in the same manner as above with a final OD_595 nm_ = 1.4 and diluted in TSB (1:200) and 200 μl/well were transferred into Corning^TM^ 96-Well Clear PVC microtiter plates. Plates were incubated at 30°C for 48 h and the microtiter plate based biofilm assay for crystal violet uptake was followed (Djordjevic et al., 2002). Eight replicates per strain were used and eight wells of TSB medium was included in the plate as control wells. Three independent experiments were performed and data expressed as mean average value ± SEM. For microscopic biofilm formation assay, sterile microscope glass slides were placed in Petri dishes and then bacterial cell suspensions were poured to ensure complete submersion of slides. Plates were incubated at 30°C for 48 h, washed in PBS, heat fixed and Gram-stained.

eDNA was isolated from bacterial biofilms with or without tunicamycin pre-treatment as before (Mann et al., 2009). Briefly, tunicamycin pretreated *L. monocytogenes* F4244 and *S. aureus* ATCC25923 cultures were seeded in wells of a 12-well tissue culture plates at 30°C for 48 h. Biofilm of each culture was gently rinsed with PBS, immersed in 0.5 ml Trizol (Thermo Fisher), and sonicated at 4°C for 15 min. Samples were removed from the wells, centrifuged at 8,000 rpm for 3 min (Eppendorf), and the eDNA from the supernatant was isolated following the manufacturer’s instruction (Thermo Fisher). The amount of total eDNA was quantified by qPCR using SYBR green (Mann et al., 2009) using primer pairs targeting 4 different genes of each *L. monocytogenes* and *S. aureus* (**Supplementary Table S1**). The amount of eDNA in biofilm was calculated by dividing the concentration of eDNA (ng/ml) by the OD_595 nm_ value of the total biofilm biomass quantified by crystal violet staining (Mann et al., 2009). Data represent relative eDNA percent compared to the untreated control.

### Effect of Tunicamycin on Bacterial Adhesion, and Invasion of Enterocyte Like-Caco-2 Cells

Caco-2 cell line (human colon adenocarcinoma cell line, ATCC, Manassas, VA, United States) were grown in 12-well tissue culture plates (Corning Life Sciences) (∼5 × 10^4^ cells) in Dulbeccos’ Modified Eagles’ Medium (DMEM with high glucose, HyClone^TM^, GE, Logan, UT, United States) with 10% (v/v) fetal bovine serum (FBS, Atlanta Biologicals, Lawrenceville, GA, United States) (DMEM-F10) at 37°C with 7% CO_2_ in a humidified cell culture incubator for 12–15 days to allow differentiation of monolayer (1 × 10^5^ cells/well) (Burkholder and Bhunia, 2010). Cell culture medium was changed twice a week.

In adhesion assay, Caco-2 cells were exposed to bacterial cells that were pre-treated with subinhibitory concentrations (0.312 μg/ml or 0.625 μg/ml for *L. monocytogenes*, and 2.5 μg/ml or 5 μg/ml for *S. aureus*) of tunicamycin or without antibiotic treatment. For preparation of bacteria, cells were washed with PBS thrice after adjusting the absorbance (600 nm) to 1 followed by resuspension in serum-free DMEM to the final concentration of 1 × 10^6^ CFU/ml to obtain an MOI of 10. Before adding the bacteria, Caco-2 cell monolayer was washed with DMEM for three times. Bacteria were added to the monolayer and incubated at 37°C with 5% CO_2_ for 1 h. The cell monolayer was rinsed with DMEM thrice. To enumerate the adhered bacterial cells, cell monolayers were treated with 0.1% Triton X-100, incubated at 37°C for 10–15 min before plating appropriate dilutions on BHI agar. Plates were incubated at 37°C for 18–24 h to enumerate CFU/ml and results were expressed as % adhesion (percent ratio of the adhered cell to the total added cells per well). Three independent experiments were performed and errors were represented as the standard error of the mean (SEM).

In invasion assay, Caco-2 cells were treated with bacteria (MOI 10) pre-treated with a subinhibitory concentration of tunicamycin or without antibiotic treatment and incubated at 37°C in 5% CO_2_ for 1 h. Caco-2 cell monolayers were washed with DMEM 3 times followed by 1 h incubation with gentamycin (50 μg/ml) in each well. The monolayers were washed with DMEM thrice. To enumerate the invading bacterial cells, cell monolayers were treated with 0.1% Triton X-100, incubated at 37°C for 10–15 min and appropriate dilutions were plated on BHI agar. Plates were incubated at 37°C for 18–24 h and bacterial cell counts were expressed as % invasion (percent ratio of invasive bacteria to the total added cells per well). Three independent experiments were performed and errors were represented as SEM.

To observe the direct effect of tunicamycin on the adhesion of *S. aureus* and *L. monocytogenes* to Caco-2 cells, bacteria and tunicamycin were inoculated simultaneously, and bacterial adhesion was enumerated by plating method as above (Burkholder and Bhunia, 2010).

### LDH and Cell Viability

To determine Caco-2 cell viability during exposure to bacteria for adhesion and invasion analysis, LDH assay was performed. After exposure of Caco-2 monolayers to bacteria for 2 h, the supernatant was collected and analyzed for lactate dehydrogenase (LDH) enzyme release. For the positive control, Caco-2 cells were treated with 1 ml of 0.1% Triton X-100 per well and only serum-free DMEM was used as the negative control. Supernatants from each treatment were transferred to the 96-well flat bottom plate in triplicates, and manufacturers (Thermo Fisher) protocol was followed to measure the LDH-release. Three independent LDH assay experiments were performed and errors were represented as the SEM.

### Luciferase Reporter Assay to Analyze NF-κB Activation

PBS-washed bacterial cells were resuspended in serum-free DMEM to the final concentration of 1 × 10^7^ CFU/ml to obtain an MOI of 10. For the cell negative control, serum-free DMEM was used. For the positive control, 0.1% LPS (*E. coli* Serotype R515, Re, TLR grade; Sigma) was added in the serum-free DMEM. The NF-κB/LUCPorter^TM^ reporter RAW 264.7 cell line (Novus Biologicals), which expresses an optimized Renilla luciferase reporter gene (RenSP) under the transcriptional control of an NF-κB response element was used (Drolia et al., 2018). The cells were seeded (1 × 10^5^ cells/well) into 96-well luminometer-compatible plates for 16 h and then treated with live bacteria for 6 h. Media from each well were aspirated, and then 100 μL of DMEM, bacteria samples or 0.1% LPS were added to each well. The plates were then incubated at 37°C for 6 h. At the end of the experiment, cells were completely lysed, and luciferase assays were performed using the Luciferase assay kit as per manufacturer instruction (Thermo Fisher). Luminescence was measured as the relative light units (RLU) using Spectramax and reported as the relative fold change compared with that of the control cells that were treated with media alone.

### Cytokine Analysis

The murine macrophage cell line, RAW 264.7 (ATCC TIB-71) was maintained in DMEM-10F. Cultures were grown at 37°C in a humidity controlled CO_2_-incubator (7% CO_2_). Adherent cells from confluent cultures were detached by 0.5% v/v trypsin at 37°C for 7 min, centrifuged (500 *g* for 3 min) and resuspended in DMEM-F10 medium to 1 × 10^5^ cells/ml. Aliquots (1 ml) were seeded in individual wells of 12-well sterile cell-culture plates and allowed to grow for 3–5 days or until confluence growth. Previously lyophilized WTA powders prepared from *S. aureus* and *L. monocytogenes* were resuspended in 1 ml deionized water and were sterilized using 0.2 μm membrane filter (Thermo Fisher). Aliquots (100 μl/well) were added and the plates were incubated at 37°C for 6, 12, and 24 h. Culture supernatants were collected, and immediately analyzed by using cytokine ELISA assay kits (eBioscience) and cytokine concentration (ng/ml) was calculated using purified cytokine standards as supplied by the manufacturer. The experiment was repeated twice in triplicate and presented as average ± SEM (Jones et al., 2005).

### Statistical Analysis

GraphPad Prism (version 6) was used to perform a one-way or a two-way analysis of variance (ANOVA) followed by a Tukey’s multiple comparisons to calculate the *P-*value for antibiotic cross-resistance, adhesion, invasion, cytotoxicity, biofilm, WTA extraction, NF-κB expression, and cytokine and immunoassay experiments performed in this study.

## Author Contributions

AB and AS: conceptualization, methodology and funding and project administration. XZ, DL, XB, RD, and ST: experimentation, data acquisition, analysis. XZ, AS, and AB: original draft generation. AB: review and editing.

## Conflict of Interest Statement

The authors declare that the research was conducted in the absence of any commercial or financial relationships that could be construed as a potential conflict of interest.

## Supplementary Material

FIGURE S1 Phase contrast microscopy of *L. monocytogenes* and *S. aureus* cells after exposure to tunicamycin. Arrows pointing to agglutinated cell mass. Magnification 1000x.

FIGURE S2 **(A)** Colony scatter patterns of *L. monocytogenes* after exposure to a different subinhibitory concentration of tunicamycin. **(B)** Enlarged images of select treatment from panel **A**. Arrows pointing to concentric rings.

FIGURE S3 Colony scatter patterns of *S. aureus* after exposure to a different subinhibitory concentration of tunicamycin.

TABLE S1 qPCR primer sets used for extracellular DNA (eDNA) analysis from biofilms.
