# Supplementary figures and images for: Tunicamycin Mediated Inhibition of Wall Teichoic Acid Affects Staphylococcus aureus and Listeria monocytogenes Cell Morphology, Biofilm Formation and Virulence
Source: Front Microbiol. 2018 Jul 2;9:1352. doi: 10.3389/fmicb.2018.01352 (PMC6043806; doi:10.3389/fmicb.2018.01352)

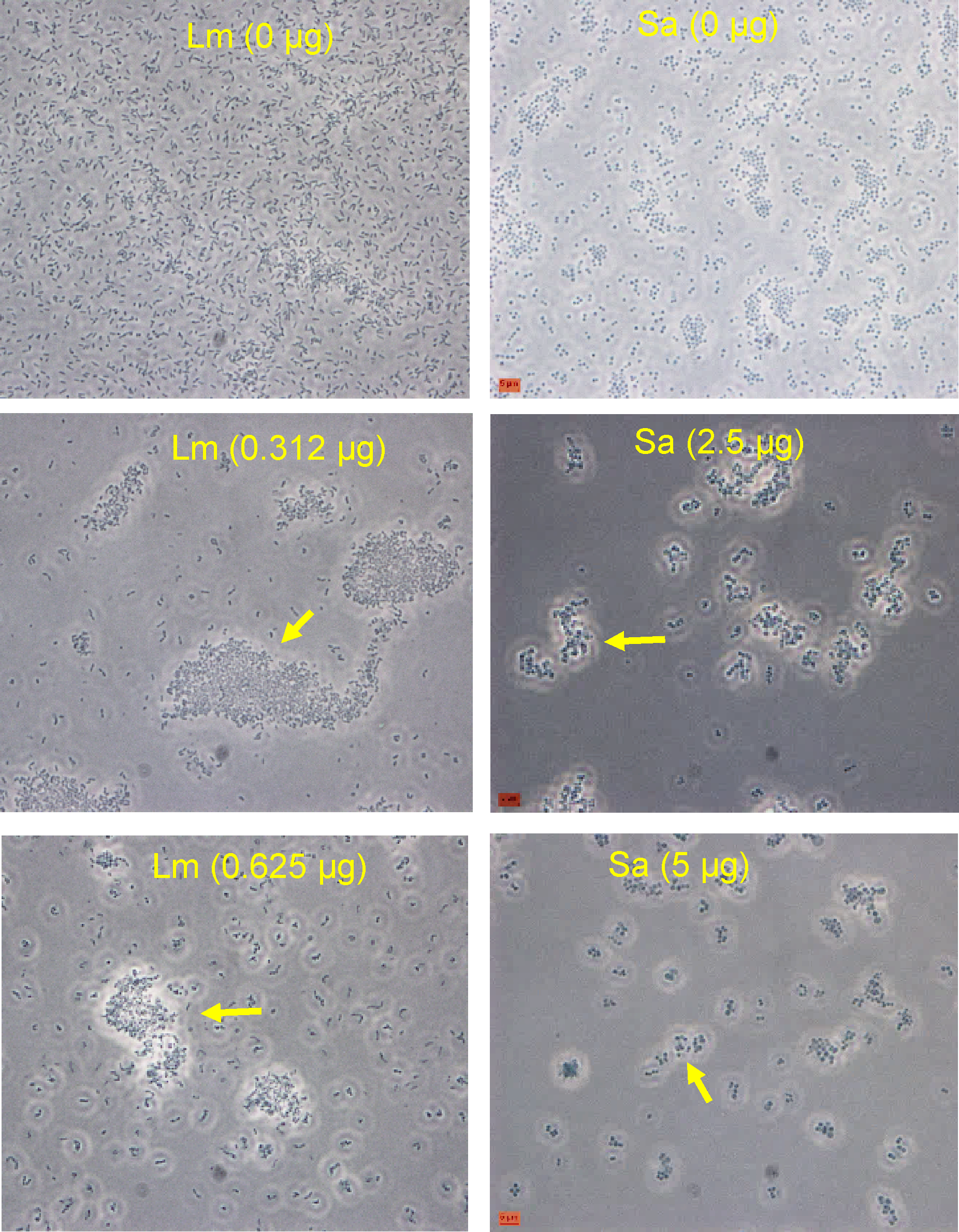

Supplement: FIGURE S1 — Phase contrast microscopy of L. monocytogenes and S. aureus cells after exposure to tunicamycin. Arrows pointing to agglutinated cell mass. Magnification 1000x. [file Image_1.TIF]

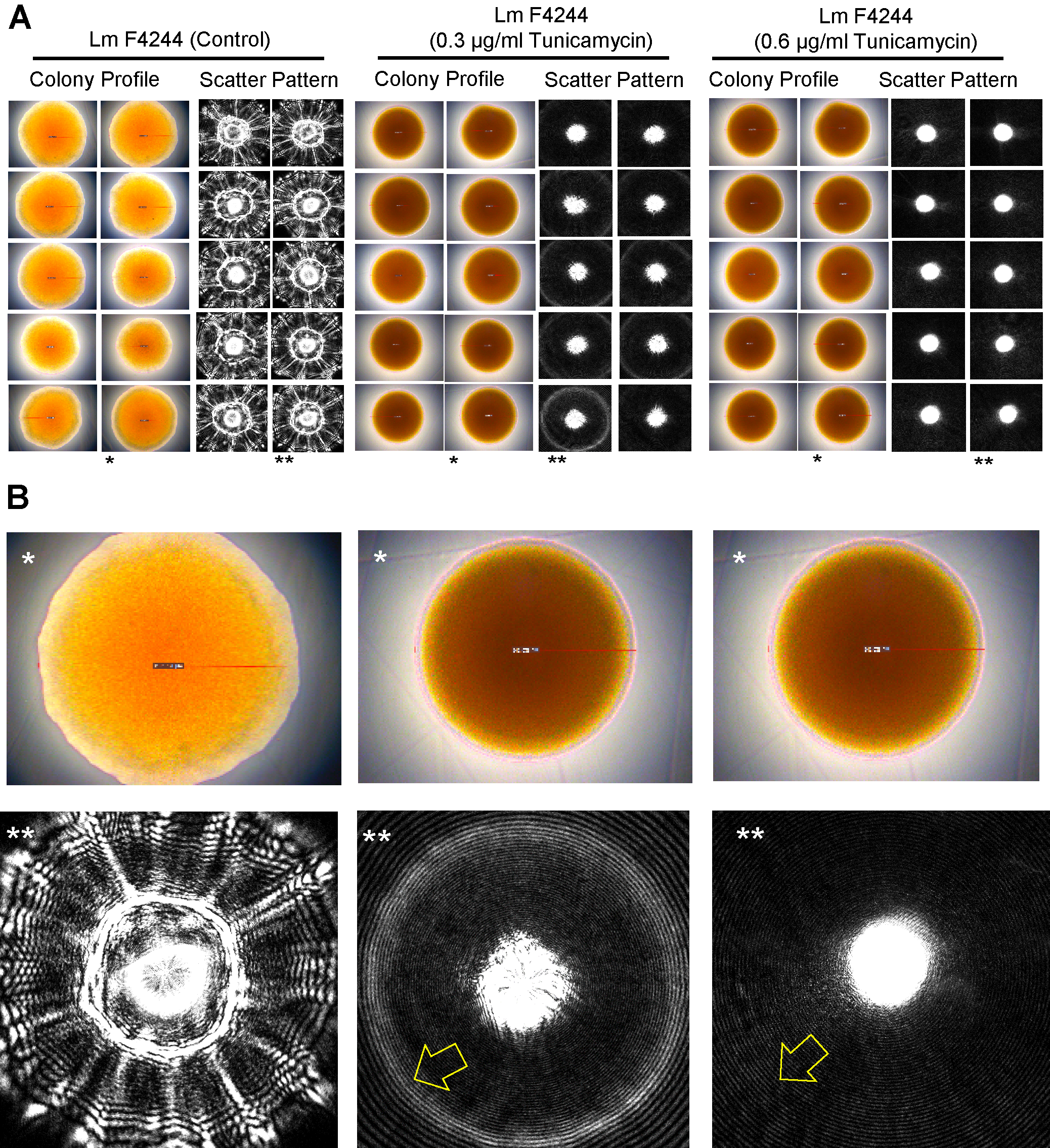

Supplement: FIGURE S2 — (A) Colony scatter patterns of L. monocytogenes after exposure to a different subinhibitory concentration of tunicamycin. (B) Enlarged images of select treatment from panel A. Arrows pointing to concentric rings. [file Image_2.TIF]

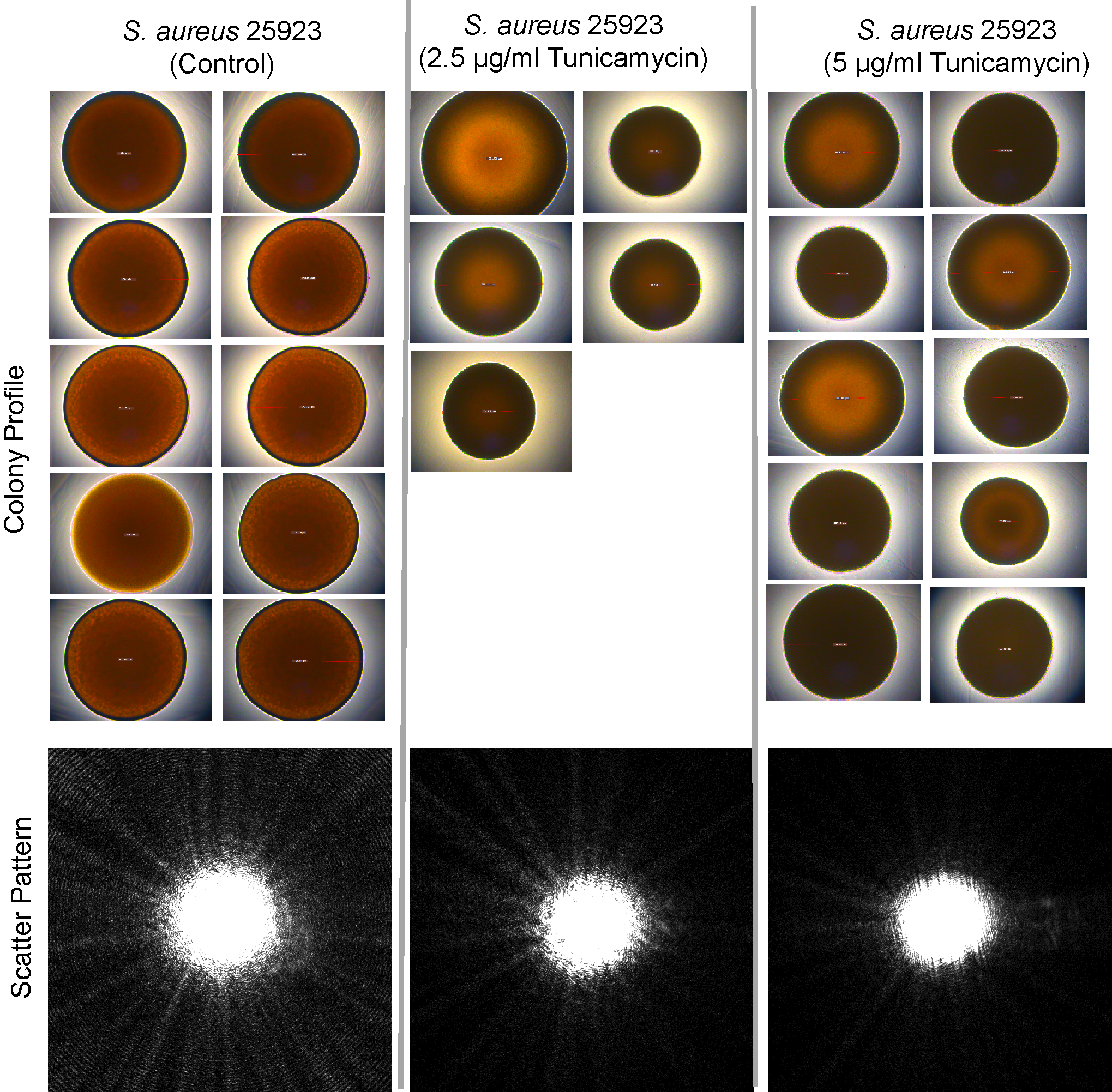

Supplement: FIGURE S3 — Colony scatter patterns of S. aureus after exposure to a different subinhibitory concentration of tunicamycin. [file Image_3.TIF]
