# Supplementary material for: Tunicamycin Mediated Inhibition of Wall Teichoic Acid Affects Staphylococcus aureus and Listeria monocytogenes Cell Morphology, Biofilm Formation and Virulence
Source: Front Microbiol. 2018 Jul 2;9:1352. doi: 10.3389/fmicb.2018.01352 (PMC6043806; doi:10.3389/fmicb.2018.01352)
Supplement: TABLE S1 — qPCR primer sets used for extracellular DNA (eDNA) analysis from biofilms. [file Table_1.DOCX]

**Supplementary Table S1.** qPCR primer sets used for extracellular DNA (eDNA) analysis from biofilms

| **Gene** | **Primer** | **Sequence** | **Source** |
| --- | --- | --- | --- |
| ***S. aureus*** |  |  |  |
| *icaA* | ICAA-F | CAACGCACTCAATCAAGGCA | This study |
|  | ICAA-R | TGGTGCATCTTGATCAACGA |  |
| *icaD* | ICAD-F | AGAGAAACAGCACTTATCGCT | This study |
|  | ICAD-R | TCGCGAAAATGCCCATAGTT |  |
| *hla* | BLA-F | TGCAATTGGTAGTCATCACGA | This study |
|  | BLA-R | TCACCAGACTTCGCTACAGT |  |
| *hlb* | BLB-F | GGGGACAATATAAACGCGCT | This study |
|  | BLB-R | ACGACCGAGTACAGGTGTTT |  |
| ***L. monocytogenes*** |  |  |  |
| *flaA* | flaA-F | ATG GCT GCT GAA ATG TCC GA | This study |
|  | flaA-R | GCG GTG TTT GGT TTG CTT GA |  |
| *lap* | qLmLAP-F | TGT TGC GAT TGT CGA TGC AC | This study |
|  | qLmLAP-R | TTT GCA AAC GCC ATA CCA GC |  |
| *inlA* | q*inlA*-F | GAACCAGCTAAGCCIGTAAAAG | ([Werbrouck et al., 2006](#_ENREF_1)) |
|  | q*inlA*-R | CGCCIGTTTGGGCATCA |  |
| *hly* | hly-F | AAA CAC GCG GAT GAA ATC GA | This study |
|  | hly-R | TAA CCT TTT CTT GGC GGC AC |  |

Werbrouck, H., Grijspeerdt, K., Botteldoorn, N., Van Pamel, E., Rijpens, N., Van Damme, J., Uyttendaele, M., Herman, L., and Van Coillie, E. (2006). Differential inlA and inlB expression and interaction with human intestinal and liver cells by *Listeria monocytogenes* strains of different origins. *Appl. Environ. Microbiol.* 72**,** 3862-3871.
